# 2,4-Dioxa-λ^6^-thia­tetra­cyclo­[5.3.1.1^5,9^.0^1,5^]dodecane-3,3-dione

**DOI:** 10.1107/S160053681202079X

**Published:** 2012-05-16

**Authors:** Savvas Ioannou, Eleni Moushi

**Affiliations:** a Chemistry Department, University of Cyprus, Nicosia 1678, Cyprus

## Abstract

The crystal structure of the title compound, C_9_H_12_O_4_S, was determined in order to investigate the effect of the eclipsed O atoms on the bond length of the vicinal quaternary C atoms. The two quaternary C atoms of the noradamantane skeleton and the two O atoms to which they are connected all located essentially in the same plane (maximum deviation = 0.01 Å), resulting in an eclipsed conformation of the C—O bonds. The C—C bond of the quaternary C atoms is 1.581 (3) Å, considerably longer than the other C—C bonds of the mol­ecule due to the stretch of the cage structure.

## Related literature

For reviews on noradamantene and analogous pyramidalized alkenes, see: Borden (1989 ▶, 1996 ▶); Vázquez & Camps (2005 ▶). For the syntheses of cyclic sulfates of acyclic alcohols, see: Byun *et al.* (2000 ▶); Kaiser (1970 ▶); Boer *et al.* (1968 ▶). For the synthesis of the precursor diol (tricyclo-[3.3.1.03,7]nonane-3,7- diol), an important inter­mediate in the synthetic route towards the generation of noradamantene, see: Zalikowski *et al.* (1980) ▶; Bertz (1985 ▶). For the synthesis of the title compound, see: Ioannou & Nicolaides (2009 ▶).
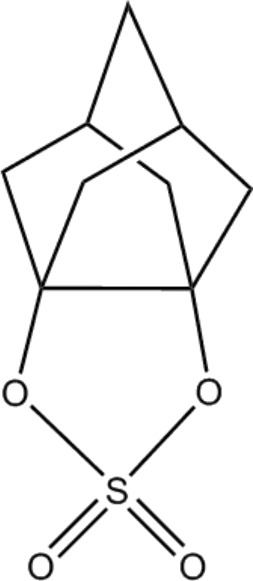


## Experimental

### 

#### Crystal data

C_9_H_12_O_4_S
*M* *_r_* = 216.25
Monoclinic, 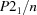

*a* = 7.6571 (3) Å
*b* = 13.0442 (6) Å
*c* = 9.1755 (4) Å
β = 95.410 (4)°
*V* = 912.37 (7) Å^3^
*Z* = 4
Mo *K*α radiation
μ = 0.34 mm^−1^
*T* = 100 K
0.05 × 0.03 × 0.02 mm

#### Data collection

Oxford Diffraction SuperNova Dual Cu at zero Atlas diffractometer
Absorption correction: multi-scan (*CrysAlis RED*; Oxford Diffraction, 2008 ▶) *T*_min_ = 0.803, *T*_max_ = 1.000
5195 measured reflections
1596 independent reflections
1389 reflections with *I* > 2σ(*I*)
*R*_int_ = 0.036

#### Refinement

*R*[*F* ^2^ > 2σ(*F* ^2^)] = 0.036
*wR*(*F* ^2^) = 0.097
*S* = 1.02
1596 reflections
127 parameters
H-atom parameters constrained
Δρ_max_ = 0.30 e Å^−3^
Δρ_min_ = −0.36 e Å^−3^

### 

Data collection: *CrysAlis CCD* (Oxford Diffraction, 2008 ▶); cell refinement: *CrysAlis RED* (Oxford Diffraction, 2008 ▶); data reduction: *CrysAlis RED*; program(s) used to solve structure: *SHELXS97* (Sheldrick, 2008 ▶); program(s) used to refine structure: *SHELXL97* (Sheldrick, 2008 ▶); molecular graphics: *DIAMOND* (Brandenburg, 2006) ▶ and *Mercury* (Macrae *et al.*, 2006 ▶); software used to prepare material for publication: *WinGX* (Farrugia, 1999 ▶) and *publCIF* (Westrip, 2010 ▶).

## Supplementary Material

Crystal structure: contains datablock(s) I, global. DOI: 10.1107/S160053681202079X/zj2070sup1.cif

Supplementary material file. DOI: 10.1107/S160053681202079X/zj2070Isup2.cdx

Structure factors: contains datablock(s) I. DOI: 10.1107/S160053681202079X/zj2070Isup3.hkl

Additional supplementary materials:  crystallographic information; 3D view; checkCIF report

## Figures and Tables

**Fig. 1.**
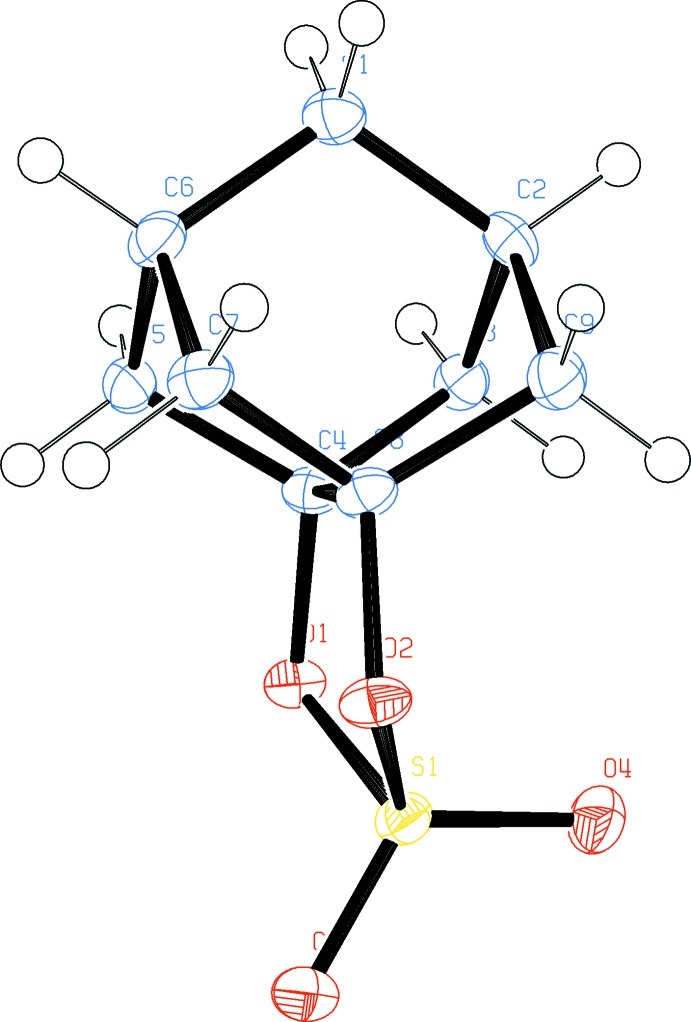
Structure of the title compound tricyclo-[3.3.1.03,7]nonane-3,7-diol cyclic sulfate with the atom-labelling. Displacement elipsoids are drawn at the 50% probability level.

**Fig. 2.**
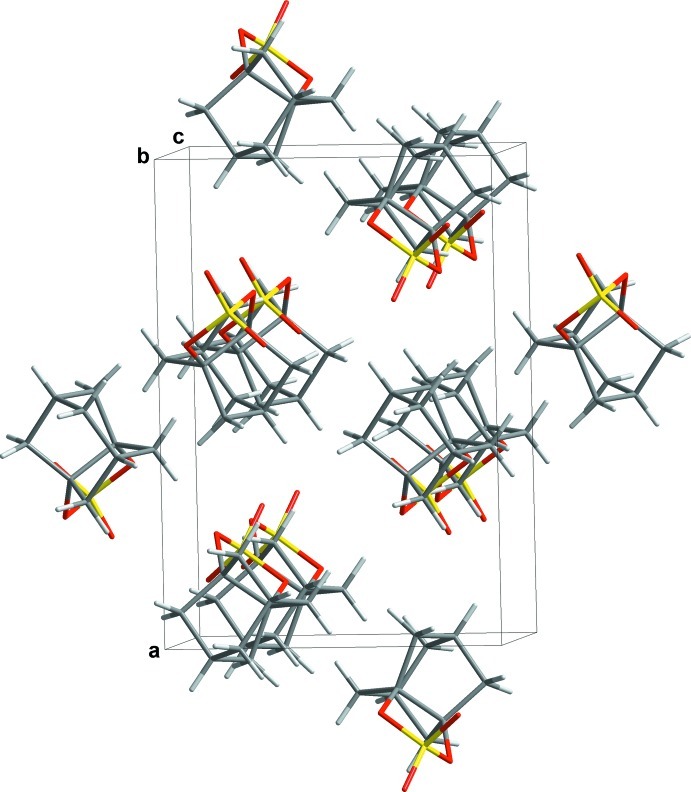
Molecular packing of the title compound, viewed along [1 0 0].

**Fig. 3.**
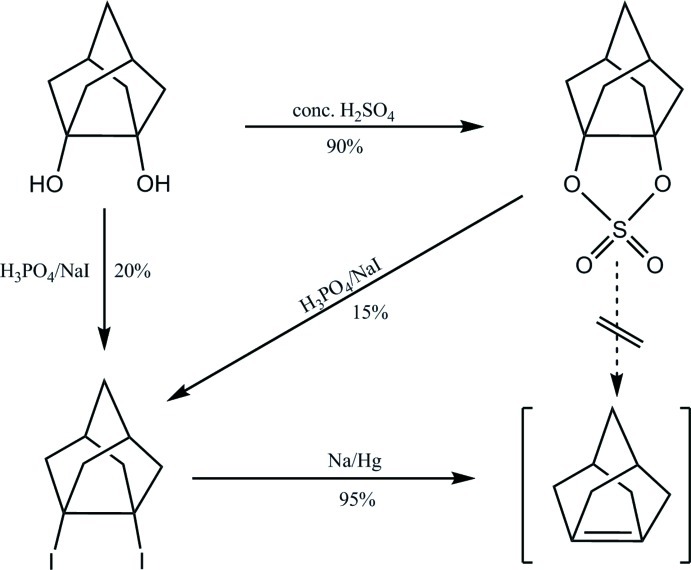
Preparation of the title compound and the experimental path of noradamantene formation.

## supplementary crystallographic information

### Comment

Five member cyclic sulfates are known for their exceptional reactivity to solvolysis in comparison to the six member rings or their acyclic analogs (Kaiser 1970, Boer *et al.* 1968). Their significant role in organic synthesis originates from their high reactivity towards various nucleophiles (Byun *et al.* 2000).

Pyramidalized alkenes is a special category of olefins which have their four substituents of the double bond not lying on the same plane (Borden 1989, 1996, Vázquez & Camps *et al.* 2005). This fact makes the higher pyramidalized alkenes (like noradamantene) very reactive and impossible to isolate at ambient conditions. Due to their high reactivity, once they form, they react instantly with any nucleophile. In the absence of any reactive compound during their formation, the most common product is their [2 + 2] dimer. Noradamantene is a member of a homologous series of this category and its preparation is quite important on studying the properties of these highly reactive compounds, as well as using it for the preparation of larger polycyclic hydrocarbons. The only convenient way of producing noradamantene quantitative is by reduction of the corresponding diiodide (scheme 3). Unfortunately, the precursor diol gives a very poor yield of diiodide (~20%) upon iodination (Ioannou *et al.* 2009). The title compound was synthesized in an attempt to build new good precursors for noradamantene, or even for the corresponding diiodide in order to improve the reaction yields.

### Experimental

Synthesis of tricyclo[3.3.1.0^3,7^]nonane-3,7-diol cyclic sulfate. Tricyclo[3.3.1.0^3,7^]nonane-3,7-diol (500 mg, 3.25 mmol) was added to concd H_2_SO_4_ (95–97%, 5 ml) and the resulting mixture was stirred at 130 ^ο^C for 1 h. After cooling, H_2_O (100 ml) was added very slowly. The solution was extracted with CH_2_Cl_2_ (4 *x* 20 ml), and the combined organic phase was dried (Na_2_SO_4_) and the solvent was removed under vacuum to give crude product (629 mg, 90%). Crystallization by slow evaporation of the solvent (hexane/dichloromethane 4:1), afforded colorless needle-like crystals. Mp 117–118 *^o^*C; *ν_max_*(KBr) 2955, 2922, 2853, 1460, 1382, 1337, 1306, 1242, 1202, 1090, 960, 837, 812, 777; *δ_H_* (300 MHz, CDCl_3_) 2.65 (2*H*, s, –CH), 2.32 (4Heq, d, J = 11.1 Hz), 2.19 (4Hax, d, J = 10.8 Hz), 1.55 (2*H*, s, –CH_2_ bridge); *δ_C_* (75.5 MHz, CDCl_3_) 94.47 (C–O), 46.44 (CH_2_), 37.04 (CH), 33.00 (CH_2_ bridge). Anal. Calcd for C_9_H_12_O_4_S: C, 50.0; H, 5.6; S, 14.8. Found: C, 50.4; H, 5.6; S, 14.4.

### Refinement

The H atoms are positioned with idealized geometry and refined using a riding model with *U*_iso_(H) = 1.2 of *U*_eq_ (C).

### Crystal data

**Table:**

C_9_H_12_O_4_S	*F*(000) = 456
*M**_r_* = 216.25	*D*_x_ = 1.574 Mg m^−^^3^
Monoclinic, *P*2_1_/*n*	Mo *K*α radiation, λ = 0.71073 Å
*a* = 7.6571 (3) Å	Cell parameters from 3034 reflections
*b* = 13.0442 (6) Å	θ = 3.1–28.8°
*c* = 9.1755 (4) Å	µ = 0.34 mm^−^^1^
β = 95.410 (4)°	*T* = 100 K
*V* = 912.37 (7) Å^3^	Needle, colorless
*Z* = 4	0.05 × 0.03 × 0.02 mm

### Data collection

**Table:**

Oxford Diffraction SuperNova Dual Cu at zero Atlas diffractometer	1596 independent reflections
Radiation source: SuperNova (Mo) X-ray Source	1389 reflections with *I* > 2σ(*I*)
Mirror monochromator	*R*_int_ = 0.036
Detector resolution: 10.4223 pixels mm^-1^	θ_max_ = 25.0°, θ_min_ = 3.1°
ω scans	*h* = −9→9
Absorption correction: multi-scan (*CrysAlis RED*; Oxford Diffraction, 2008)	*k* = −14→15
*T*_min_ = 0.803, *T*_max_ = 1.000	*l* = −10→10
5195 measured reflections	

### Refinement

**Table:**

Refinement on *F*^2^	Primary atom site location: structure-invariant direct methods
Least-squares matrix: full	Secondary atom site location: difference Fourier map
*R*[*F*^2^ > 2σ(*F*^2^)] = 0.036	Hydrogen site location: inferred from neighbouring sites
*wR*(*F*^2^) = 0.097	H-atom parameters constrained
*S* = 1.02	*w* = 1/[σ^2^(*F*_o_^2^) + (0.0463*P*)^2^ + 0.8407*P*] where *P* = (*F*_o_^2^ + 2*F*_c_^2^)/3
1596 reflections	(Δ/σ)_max_ < 0.001
127 parameters	Δρ_max_ = 0.30 e Å^−^^3^
0 restraints	Δρ_min_ = −0.36 e Å^−^^3^

### Special details

**Table:**

Geometry. All e.s.d.'s (except the e.s.d. in the dihedral angle between two l.s. planes) are estimated using the full covariance matrix. The cell e.s.d.'s are taken into account individually in the estimation of e.s.d.'s in distances, angles and torsion angles; correlations between e.s.d.'s in cell parameters are only used when they are defined by crystal symmetry. An approximate (isotropic) treatment of cell e.s.d.'s is used for estimating e.s.d.'s involving l.s. planes.
Refinement. Refinement of *F*^2^ against ALL reflections. The weighted *R*-factor *wR* and goodness of fit *S* are based on *F*^2^, conventional *R*-factors *R* are based on *F*, with *F* set to zero for negative *F*^2^. The threshold expression of *F*^2^ > σ(*F*^2^) is used only for calculating *R*-factors(gt) *etc*. and is not relevant to the choice of reflections for refinement. *R*-factors based on *F*^2^ are statistically about twice as large as those based on *F*, and *R*- factors based on ALL data will be even larger.

### Fractional atomic coordinates and isotropic or equivalent isotropic displacement parameters (Å^2^)

**Table:**

	*x*	*y*	*z*	*U*_iso_*/*U*_eq_	
S1	−0.10031 (6)	0.20071 (4)	0.77561 (6)	0.01630 (19)	
O1	−0.05353 (18)	0.13375 (11)	0.64114 (16)	0.0169 (4)	
O2	0.09088 (18)	0.23740 (11)	0.83001 (17)	0.0175 (4)	
O3	−0.20095 (19)	0.28604 (12)	0.72290 (18)	0.0224 (4)	
O4	−0.16597 (19)	0.13680 (12)	0.88353 (17)	0.0226 (4)	
C1	0.4547 (3)	0.01132 (17)	0.7302 (2)	0.0185 (5)	
H1A	0.5641	0.0202	0.7913	0.022*	
H1B	0.4684	−0.0465	0.6658	0.022*	
C2	0.3060 (3)	−0.01186 (16)	0.8282 (2)	0.0177 (5)	
H2	0.3305	−0.0733	0.8881	0.021*	
C3	0.1296 (3)	−0.01977 (16)	0.7334 (2)	0.0170 (5)	
H3A	0.1327	−0.0705	0.6564	0.020*	
H3B	0.0330	−0.0344	0.7914	0.020*	
C4	0.1219 (3)	0.08940 (16)	0.6732 (2)	0.0147 (5)	
C5	0.2365 (3)	0.09867 (17)	0.5474 (2)	0.0178 (5)	
H5A	0.2076	0.1589	0.4879	0.021*	
H5B	0.2300	0.0380	0.4860	0.021*	
C6	0.4163 (3)	0.10886 (17)	0.6370 (2)	0.0180 (5)	
H6	0.5104	0.1242	0.5750	0.022*	
C7	0.3780 (3)	0.19974 (17)	0.7360 (3)	0.0181 (5)	
H7A	0.4735	0.2121	0.8111	0.022*	
H7B	0.3524	0.2620	0.6803	0.022*	
C8	0.2171 (3)	0.15760 (16)	0.7992 (2)	0.0153 (5)	
C9	0.2700 (3)	0.08211 (17)	0.9218 (2)	0.0189 (5)	
H9A	0.1757	0.0698	0.9831	0.023*	
H9B	0.3742	0.1043	0.9819	0.023*	

### Atomic displacement parameters (Å^2^)

**Table:**

	*U*^11^	*U*^22^	*U*^33^	*U*^12^	*U*^13^	*U*^23^
S1	0.0148 (3)	0.0156 (3)	0.0191 (3)	0.0000 (2)	0.0042 (2)	−0.0004 (2)
O1	0.0144 (7)	0.0174 (8)	0.0187 (8)	0.0018 (6)	0.0006 (6)	−0.0029 (6)
O2	0.0150 (7)	0.0138 (8)	0.0239 (9)	0.0009 (6)	0.0027 (6)	−0.0052 (7)
O3	0.0209 (8)	0.0200 (9)	0.0269 (10)	0.0054 (7)	0.0048 (7)	0.0023 (7)
O4	0.0223 (8)	0.0237 (9)	0.0229 (9)	−0.0025 (7)	0.0076 (6)	0.0031 (7)
C1	0.0188 (11)	0.0159 (11)	0.0210 (12)	0.0020 (9)	0.0026 (9)	−0.0014 (9)
C2	0.0210 (11)	0.0120 (11)	0.0196 (12)	0.0014 (9)	0.0005 (9)	0.0040 (9)
C3	0.0184 (11)	0.0132 (11)	0.0196 (12)	−0.0017 (9)	0.0037 (8)	−0.0004 (9)
C4	0.0116 (10)	0.0132 (11)	0.0191 (12)	−0.0001 (9)	−0.0001 (8)	−0.0010 (9)
C5	0.0209 (11)	0.0169 (11)	0.0159 (12)	0.0022 (9)	0.0036 (9)	0.0005 (9)
C6	0.0165 (10)	0.0161 (11)	0.0225 (12)	−0.0006 (9)	0.0075 (9)	0.0004 (9)
C7	0.0156 (11)	0.0155 (12)	0.0236 (13)	−0.0016 (9)	0.0037 (9)	−0.0011 (9)
C8	0.0148 (10)	0.0124 (11)	0.0189 (12)	0.0007 (9)	0.0037 (8)	−0.0035 (9)
C9	0.0185 (10)	0.0222 (12)	0.0158 (12)	0.0001 (10)	0.0007 (8)	0.0006 (10)

### Geometric parameters (Å, º)

**Table:**

S1—O3	1.4129 (16)	C3—H3B	0.9700
S1—O4	1.4221 (16)	C4—C5	1.520 (3)
S1—O2	1.5759 (15)	C4—C8	1.581 (3)
S1—O1	1.5801 (15)	C5—C6	1.541 (3)
O1—C4	1.466 (2)	C5—H5A	0.9700
O2—C8	1.466 (2)	C5—H5B	0.9700
C1—C6	1.546 (3)	C6—C7	1.538 (3)
C1—C2	1.546 (3)	C6—H6	0.9800
C1—H1A	0.9700	C7—C8	1.514 (3)
C1—H1B	0.9700	C7—H7A	0.9700
C2—C9	1.536 (3)	C7—H7B	0.9700
C2—C3	1.540 (3)	C8—C9	1.521 (3)
C2—H2	0.9800	C9—H9A	0.9700
C3—C4	1.526 (3)	C9—H9B	0.9700
C3—H3A	0.9700		
			
O3—S1—O4	118.88 (9)	C3—C4—C8	105.15 (17)
O3—S1—O2	109.24 (9)	C4—C5—C6	98.78 (17)
O4—S1—O2	109.66 (9)	C4—C5—H5A	112.0
O3—S1—O1	108.94 (9)	C6—C5—H5A	112.0
O4—S1—O1	109.92 (9)	C4—C5—H5B	112.0
O2—S1—O1	98.22 (8)	C6—C5—H5B	112.0
C4—O1—S1	109.39 (12)	H5A—C5—H5B	109.7
C8—O2—S1	109.44 (12)	C7—C6—C5	99.84 (16)
C6—C1—C2	111.76 (17)	C7—C6—C1	110.17 (18)
C6—C1—H1A	109.3	C5—C6—C1	109.73 (18)
C2—C1—H1A	109.3	C7—C6—H6	112.2
C6—C1—H1B	109.3	C5—C6—H6	112.2
C2—C1—H1B	109.3	C1—C6—H6	112.2
H1A—C1—H1B	107.9	C8—C7—C6	98.85 (17)
C9—C2—C3	100.12 (16)	C8—C7—H7A	112.0
C9—C2—C1	110.47 (17)	C6—C7—H7A	112.0
C3—C2—C1	109.83 (18)	C8—C7—H7B	112.0
C9—C2—H2	111.9	C6—C7—H7B	112.0
C3—C2—H2	111.9	H7A—C7—H7B	109.7
C1—C2—H2	111.9	O2—C8—C7	113.03 (17)
C4—C3—C2	98.27 (16)	O2—C8—C9	116.85 (17)
C4—C3—H3A	112.1	C7—C8—C9	110.41 (17)
C2—C3—H3A	112.1	O2—C8—C4	105.88 (15)
C4—C3—H3B	112.1	C7—C8—C4	105.08 (17)
C2—C3—H3B	112.1	C9—C8—C4	104.40 (16)
H3A—C3—H3B	109.8	C8—C9—C2	98.76 (17)
O1—C4—C5	113.56 (17)	C8—C9—H9A	112.0
O1—C4—C3	116.37 (16)	C2—C9—H9A	112.0
C5—C4—C3	110.08 (18)	C8—C9—H9B	112.0
O1—C4—C8	105.98 (16)	C2—C9—H9B	112.0
C5—C4—C8	104.54 (16)	H9A—C9—H9B	109.7
